# The Association of vitamin D status and fasting glucose according to body fat mass in young healthy Thais

**DOI:** 10.1186/1472-6823-13-60

**Published:** 2013-12-27

**Authors:** Hataikarn Nimitphong, La-or Chailurkit, Suwannee Chanprasertyothin, Piyamitr Sritara, Boonsong Ongphiphadhanakul

**Affiliations:** 1 Faculty of Medicine, Ramathibodi Hospital, Mahidol University, Rama 6 Rd., Rajthevi, Bangkok 10400, Thailand

**Keywords:** 25-hydroxyvitamin D, Fasting plasma glucose, Total body fat mass, Adiposity

## Abstract

**Background:**

Existing inconclusive data on the relationship between vitamin D status and human glucose homeostasis suggests that other factors, such as adiposity, might influence this relationship. The present study aimed to investigate the association between 25-hydroxyvitamin D [25(OH)D] and fasting plasma glucose (FPG) in the context of different amounts of total body fat in a healthy community-based population in Bangkok, Thailand.

**Methods:**

This cross-sectional study was a part of health survey of employees of the Electricity Generating Authority of Thailand. There were 1,990 healthy subjects (72.8% male) in this study. Total body fat was measured by bioelectrical impedance analysis. Total serum 25(OH)D, 25(OH)D_3_ and 25(OH)D_2_ were measured by LC-MS/MS.

**Result*s*:**

Age (r = 0.134, *p* < 0.001) and FPG (r = 0.089, *p* < 0.001) were positively correlated with 25(OH)D levels, while total body fat mass (r = -0.049, *p* = 0.03) were negatively correlated with 25(OH)D levels. 25(OH)D levels were higher in males than in females (65.0 ± 0.5 vs. 53.5 ± 0.5 nmol/L, *p* < 0.001). After controlling for age, gender and total fat mass, FPG was no longer correlated with 25(OH)D. However, when subjects were stratified according to fat-free mass tertiles and controlled for age and gender, there was a positive, although weak association between 25(OH)D levels and FPG (*p* = 0.01) in the lowest tertile.

**Conclusions:**

We therefore speculate that adiposity might influence the relationship of vitamin D status and FPG.

## Background

In addition to its established role in calcium and bone metabolism, vitamin D possesses numerous other biological functions [1,2]. With regard to glucose homeostasis, it has been shown that vitamin D affects pancreatic beta-cell proliferation and survival [3,4]. It has also been demonstrated that vitamin D improves insulin sensitivity [5,6]. At the population level, several association studies have demonstrated a relationship between impaired vitamin D status and higher risk of prevalence as well as incidence of diabetes [7-9], although these findings are not undisputed. We found in a recent study of subjects from the 4^th^ Thai National Health Examination Survey (2,641 adults, aged 15–98) that low 25(OH)D_3_ but not 25(OH)D_2_ level was significantly associated with increased odds of diabetes only in the subgroup of urban elderly (≥70 years old) [10]. It is thus likely that if vitamin D does indeed affect glucose homeostasis, its influence is probably minute and there may be other interacting factors, for example body mass index (BMI), which cause results from population studies to be less consistent. Studies in vitamin D receptor (VDR) knockout mice revealed a lean phenotype resistant to diet-induced obesity [11,12], suggesting that vitamin D may have a role in promoting adipose tissue development in an *in vivo* context. Because low vitamin D status is associated with human obesity [13-15], it is possible that vitamin D action in adipose tissue underlies the relationship between vitamin D and glucose homeostasis in humans. The aim of this study is to explore the relationship between 25(OH)D, a marker of vitamin D status, and fasting plasma glucose (FPG) in the context of different amounts of total body fat in a healthy community-based population in Bangkok, Thailand. The secondary objective is to investigate the association between 25(OH)D and FPG in subgroups of subjects stratified by gender and age.

## Methods

This study was part of a health survey of 1,990 employees of the Electricity Generating Authority of Thailand (EGAT). Institutional Review Board approval was obtained prior to the commencement of the study, and all subjects gave informed consent. Described in detail elsewhere [16], survey data was collected by using self-administered questionnaires, physical examinations, electrocardiography, chest radiography, and blood analysis. Anthropometric variables including weight, height and waist circumferences were measured using standard techniques in all subjects [17]. Waist circumference was measured at the midway between the last rib and the iliac crest [17]. BMI was derived by weight (kg)/height(m)^2^. Body composition was determined after at least 3 h of fasting using multifrequency bioelectrical impedance analysis with eight-point tactile electrodes (InBody 720; Biospace, Seoul, Korea). Fasting blood samples were obtained and sent for laboratory analysis of fasting plasma glucose (FPG) and 25(OH)D measurements. The study was approved by the IRB of Ramathibodi hospital, Mahidol University.

### Serum 25-hydroxyvitamin D [25(OH)D] measurement

Serum 25(OH)D_2_ and 25(OH)D_3_ were analyzed by LC-MS/MS with an Agilent 1200 Infinity liquid chromatograph (Agilent Technologies, Waldbronn, Germany) coupled to a QTRAP^®^ 5500 tandem mass spectrometer (AB SCIEX, Framingham MA, USA) using a MassChrom^®^ 25-OH-Vitamin D_3_/D_2_ diagnostics kit (ChromSystems, Munich, Germany). The summation of serum 25(OH)D_2_ and 25(OH)D_3_ [total 25(OH)D] was used to reflect vitamin D status. Vitamin D deficiency was defined as having 25(OH)D levels of less than 50 nmol/L [18]. The inter-assay and intra-assay coefficients of variation of total serum 25(OH)D level were 6.3% and 5.0%, respectively.

### Statistical analysis

Data were expressed as mean ± standard error of the mean (SEM). All data were normally distributed. Differences between two groups were assessed by Student’s *t*-test. The correlations between dependent and independent variables were tested with Pearson correlation. Multiple linear regression analysis was performed to identify the association between FPG (the dependent variable) and 25(OH)D in each subgroup, stratified by gender (male vs. female), age (25–34, 35–44 and 45–54 years) and tertile of total body fat. A *p* value less than 0.05 was considered statistically significant. All analyses were performed using the SPSS statistical software package, version 17.0 (SPSS Inc., Chicago IL, USA).

## Results

Table 1 demonstrates the clinical characteristics of the study population. Subjects were mostly males (n = 1,449, 72.8%) because of the demographic structure of EGAT. A data comparison between males and females revealed that males were slightly older, had significantly higher BMI, waist circumference (WC), fasting plasma glucose (FPG) and muscle mass, and significantly lower total body fat mass.

**Table 1:** Clinical characteristics of the study population

**Parameters**	**Male n = 1,449**	**Female n = 541**	***P *** **value**
Age (years)	40.1 ± 0.2 (25–54)	39.5 ± 0.3 (25–54)	0.05
BMI (kg/m^2^)	24.5 ± 0.1 (15–48)	22.0 ± 0.2 (15–39)	<0.001
Waist circumference (WC) (cm)	88.8 ± 0.2 (54–149)	78.0 ± 0.4 (57–112)	<0.001
Fasting plasma glucose (FPG) (mmol/L)	5.2 ± 0.0 (4–17.8)	4.9 ± 0.0 (3.7–17.2)	<0.001
Muscle mass (kg)	29.4 ± 0.1 (18.4–44.4)	19.7 ± 0.1 (13.4–29.0)	<0.001
Total body fat mass (kg)	17.6 ± 0.2 (2.9–68.1)	18.3 ± 0.3 (6.8–46.4)	0.04
Percent body fat (%)	24.5 ± 0.2 (6.7–46.7)	32.5 ± 0.3 (16.9–51.1)	<0.001
Vitamin D status			
<50 nmol/L (n = 433)	44 ± 0.5 (n = 201)	42 ± 0.5 (n = 233)	0.001
≥50 nmol/L (n = 1,592)	68.5 ± 0.5 (n = 1,248)	62.2 ± 0.5 (n = 308)	<0.001

Data is expressed as mean ± SEM (range).

Body composition was assessed by bioelectrical impedance analysis (BIA).

With regard to vitamin D status, mean total 25(OH)D concentrations were significantly higher in males than in females (65.0 ± 0.5 vs. 53.5 ± 0.5 nmol/L, *P* < 0.001). The dominant form of total 25(OH)D in this population group was 25(OH)D_3_. As expected, females had a higher prevalence of vitamin D deficiency: 43.1% of females had 25(OH)D less than 50 nmol/L, whereas 13.9% of males were classified as vitamin D deficient (Table 1). Only 3 of subjects, 1 male and 2 females, had 25(OH)D levels less than 25 nmol/L.

**Figure 1:**
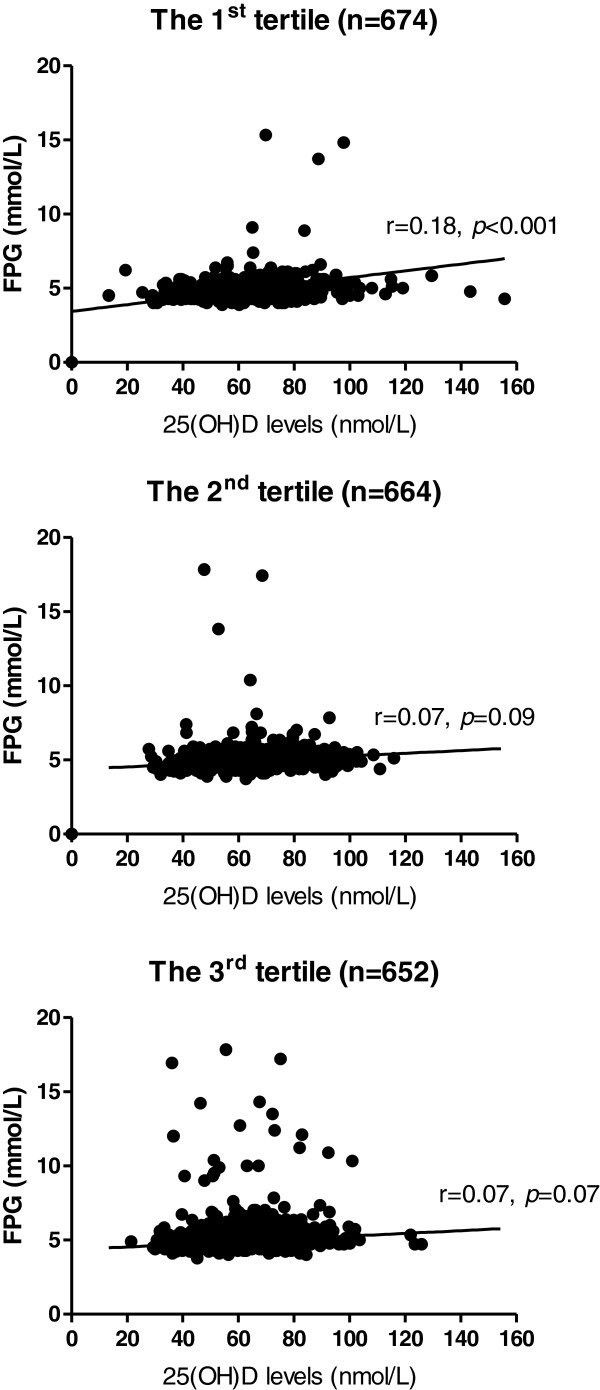
Scatter plots of fasting plasma glucose and total 25(OH)D according to body fat mass tertiles.

Univariate analyses revealed that age (r = 0.134, *p* < 0.001), FPG (r = 0.089, *p* < 0.001) and total body fat mass (r = -0.049, *p* = 0.027) were significantly, although weakly correlated with 25(OH)D levels. When classified subjects into 3 groups according to fat mass tertiles, a positive association between 25(OH)D and FPG was consistently found across 3 groups, but reached statistically significance only in the lowest tertile group (Figure 1). Multiple linear regression analysis showed that age, gender and total body fat mass, but not 25(OH)D, were independently correlated with FPG. However, when subjects were stratified according to fat mass tertiles and that the analysis was controlled for age and gender, it was found that there was a significant association (*p* = 0.01), although a weak positive correlation (r = 0.097), between 25(OH)D levels and FPG only in the lowest tertile group, as shown in Table 2. Nevertheless, no relationship between 25(OH)D and FPG was detected when subjects were stratified according to age group (controlled for gender and body fat mass) or gender (controlled for age and body fat mass) (Table 2).

**Table 2:** Adjusted association of serum FPG and 25(OH)D stratified by gender, age range and tertile of total body fat

**Parameters**	**25(OH)D levels (nmol/L)**	**Standardized coefficients (β)**	***P *** **value**
**Gender**^**1**^			
Male (n = 1,449)	65 ± 0.5	0.031	0.25
Female (n = 541)	53.5 ± 0.5	0.042	0.35
**Age**^**2**^ [n, range of age (years)]			
group 1 (n = 402, age 25–34)	58.5 ± 0.8	0.023	0.64
group 2 (n = 1,064, age 35–44)	62.5 ± 0.5	0.044	0.13
group 3 (n = 524, age 44–54)	63.8 ± 0.8	0.039	0.36
**Total body fat mass**^**3**^ [mean ± SEM; range (kg)]			
1^st^ tertile (n = 674) (11.5 ± 0.1; 2.9–14.5)	62.5 ± 0.8	0.097	0.01
2^nd^ tertile (n = 664) (17.0 ± 0.1; 14.6–19.5)	62.3 ± 0.5	-0.013	0.73
3^rd^ tertile (n = 652) (25.1 ± 0.2; 19.6–68.1)	61 ± 0.5	0.025	0.51

Data is expressed as mean ± SEM.

^1^controlled for age and body fat mass; ^2^controlled for gender and body fat mass; ^3^controlled for age and gender.

## Discussion

There is increasing evidence that vitamin D status may modify the risk of type 2 diabetes. Most of the studies looking into the relationship between 25(OH)D levels or vitamin D supplementation and FPG, glucose homeostasis or incidence of new onset of type 2 diabetes have been performed in Caucasian populations [7-9]. The difference of this relationship between Caucasians and Asians has not been well recognized. In the present study, no association was demonstrated between vitamin D status and FPG. The same finding was reported in a study of 380 Malay adults (mean age of 48.5 years; 42% male). Investigators classified subjects into two groups, vitamin D insufficient [25(OH)D levels <50 nmol/L] and vitamin D sufficient [25(OH)D levels ≥50 nmol/L], and found no correlation between 25(OH)D levels and FPG [19]. On the other hand, in the Fourth Korea National Health and Nutrition Examination Survey (n = 5,787; mean age of 48.5 (male) and 49.1 (female) years; 42% male), 25(OH)D levels were inversely associated with FPG after adjusting for age and sex [20]. This was in accordance with the findings in a study of 3,262 elderly Chinese (age 50–70 years; 44% male), where 25(OH)D levels were negatively associated with FPG after controlling for age, sex and BMI [21]. Differences in study results can be partly explained by different subject characteristics, i.e. the subjects in our study were relatively young and predominantly male.

In this study, we also confirmed a negative correlation between 25(OH)D and degree of adiposity as defined by BMI, WC (data not shown) and total body mass. The predominant source of vitamin D in these subjects was from sun exposure. It has been reported that with a similar amount of 7-dehydrocholesterol in the epidermis and using a similar amount of UVB irradiation, serum vitamin D_3_ levels of obese subjects were 50% lower than those of non-obese subjects; this suggested the possibility of sequestration of vitamin D in expanded adipose tissue [22]. In a hypocaloric intervention study, with 10% weight loss 25(ΟΗ)D levels increased by 34% without any changes in vitamin D intake [23]. It is also conceivable that reducing fat mass may somehow influence the metabolism of vitamin D and some of its effects.

In contrast to the results of a previous study in Thailand [10], which demonstrated an association between lower vitamin D status and an increased risk of diabetes in subgroup of older subjects residing in urban areas, the present study reported the reversed direction of the relationship in the younger age group. A statistical interaction was also demonstrated between body fat mass, vitamin D status and FPG. In the lowest tertile of body fat mass subgroups, the higher the participants’ 25(OH)D levels, the higher their FPG; this was in contrast to previous studies in both Caucasians and Asians [20,21], where the results showed an opposite trend. Although the strength of the statistical association was relatively weak and the underlying basis unclear, the finding is intriguing. In spite of the fact that most epidemiologic studies demonstrated lower circulating vitamin D is generally associated with increased adiposity [24], underlined mechanism of the association has not been well established. Nevertheless, the mechanistic studies of this correlation in mouse model are complex. For example VDR knockout mice were lean [11]. The same study also demonstrated an increase in uncoupling protein (UCP), a marker of brown adipose tissue [11]. The main function of brown adipose tissue is to regulate thermogenesis by expressing UCPs that separate oxidative phosphorylation from ATP (adenosine triphosphate) production, producing heat in place of ATP [25]. Confirmatory data from a recent study of overexpression of VDR in mice demonstrated an increase in fat mass, mainly due to markedly reduced energy expenditure. In addition, the expression of genes involved in the regulation of fatty acid transport, thermogenesis and lipolysis were suppressed in the transgenic mice [25]. Taken together, these data confirm an important role of VDR in the regulation of energy metabolism. Brown adipose tissue has recently been confirmed to be functionally active in human adults [26], and is likely to decrease with age [26]. It is therefore conceivable that in younger adults, who may possess a higher amount of brown adipose tissue, vitamin D may have a greater influence in inhibiting the function of brown adipose tissue rather than in inhibiting lipogenesis and its metabolic consequences in white adipose tissue. Further studies to explore the comparative effects of vitamin D on brown and white adipose tissues are warranted.

If vitamin D does indeed increase FPG, it remains unclear whether it would increase diabetes risk and diabetic complications. Although FPG is generally used for the diagnosis of diabetes [27], it has been recognized that its relationship to diabetic complications may not be straightforward. For example, MODY (maturity onset diabetes of the young) patients with mutation in the glucokinase gene presented with elevated FPG but generally without a significant increase in average HbA1c and diabetes-related complications [28]. Further studies to clarify the issue, using measures more directly related to diabetes complications, are therefore warranted. One of the limitation of this study is we could only assess FPG (single measurement) and did not examine other metabolic measurement such as HbA1c, fasting insulin, lipid profile. We did not have data about previous history of diabetes or metabolic syndrome in our study subjects. Nonetheless, statistical analysis which including or excluding subjects who had FPG ≥ 7 mmol/L (126 mg/dL) provided the same results. In addition, other measures that can potentially affect both diabetic risk and vitamin D such as calcium were not evaluated.

## Conclusion

In conclusion our findings were contrary to those of most studies, where a relationship between vitamin D status and diabetes has been demonstrated. Although the present findings need to be replicated in separate populations, they suggest the heterogeneity of the effect of vitamin D status based on interacting factors such as body fat mass; this may underlie the inconsistent results among previously performed studies.

## Competing interests

The authors affirm they have no competing interests.

## Authors’ contributions

HN and BO conceived of the study, participated in its design and coordination, performed the statistical analysis and helped to draft the manuscript. LC carried out the vitamin D metabolites measurement (LC-MS/MS). SC carried out the biochemical measurement. PS participated in its design and coordination. All authors read and approved the final manuscript.

## Pre-publication history

The pre-publication history for this paper can be accessed here:

http://www.biomedcentral.com/1472-6823/13/60/prepub
